# Pathways from childhood trauma to suicidal ideation: mediating through difficulties in emotion regulation and depressive symptoms

**DOI:** 10.1186/s12888-023-04699-8

**Published:** 2023-04-28

**Authors:** Marziyeh Laghaei, Mahnaz Mehrabizadeh Honarmand, Laura Jobson, Hamed Abdollahpour Ranjbar, Mojtaba Habibi Asgarabad

**Affiliations:** 1 Department of Psychology, Shahid Chamran University of Ahvaz, Ahvaz, Iran; 2 School of Psychological Sciences and Turner Institute for Brain and Mental Health, Monash University, Melbourne, Australia; 3 Department of Psychology, College of Social Sciences and Humanities, Koç University, Istanbul, Turkey; 4 Health Promotion Research Center, Iran University of Medical Sciences, Tehran, Iran; 5 Department of Psychology, Norwegian University of Science and Technology, Dragvoll, 7491 Trondheim, Norway; 6 Department of Health Psychology, School of Behavioral Sciences and Mental Health (Tehran Institute of Psychiatry), Iran University of Medical Sciences, Tehran, Iran; 7 Positive Youth Development Lab, Human Development & Family Sciences, Texas Tech University, Texas, USA; 8 Center of Excellence in Cognitive Neuropsychology, Institute for Cognitive and Brain Sciences, Shahid Beheshti University, Tehran, Iran

**Keywords:** Suicidal ideation, Depressive symptoms, Emotion regulation difficulties, Childhood trauma

## Abstract

**Objective:**

Suicidal ideation is a clinical exigency heightening the risk of suicide at different levels of suicidal behavior. This study sought to explore crucial correlates of this phenomenon with a structural equation modeling approach. Accordingly, the mediating role of depressive symptoms and difficulties in emotion regulation between childhood trauma and suicidal ideation were explored.

**Method:**

The sample consisted of 372 university students (57.3% females, M = 20.75, SD = 2.25) who completed self-report measures examining experiences of childhood trauma, depressive symptoms, difficulties in emotion regulation, and suicidal ideation. Structural equation modeling was employed, and mediation analysis was conducted. Confirmatory factor analysis was used to test the measurement model of each construct before evaluating the conceptual mediated model.

**Results:**

Findings indicate that depressive symptoms with difficulties in emotion regulation had the strongest association (*r* = 0.60, *P* = 0.001), then depressive symptoms and suicidal ideation (*r* = 0.58, *P* = 0.001), suicidal ideation with difficulties in emotion regulation (*r* = 0.45, *P* = 0.001) then suicidal ideation with childhood trauma (*r* = 0.39, *P* = 0.001), difficulties in emotion regulation with childhood trauma (*r* = 0.36 *P* = 0.001) and finally depressive symptoms and childhood trauma (*r* = 0.35, *P* = 0.001). Regarding indirect paths, difficulties in emotion regulation and depression function together (in a sequential path) to mediate the association between childhood trauma and suicidal ideation ( *χ*^*2*^(68) = 216.86, *P* < 0.01, CFI = 0.95, TLI = 0.93, RMSE = 0.077, CI [0.066 to 0.089], SRMR = 0.049).

**Conclusion:**

Results demonstrate that childhood trauma, depressive symptoms, and difficulties in emotion regulation are linked to suicidal ideation, highlighting the necessity of recognizing and addressing suicidal ideation as well as the factors that contribute to suicidal ideation. Emotion regulation interventions can be effective in reducing the negative effects of childhood trauma and lowering the risk of suicide. These interventions can help in reducing depressive symptoms and improve overall mental well-being, leading to a lower risk of suicide.

**Supplementary Information:**

The online version contains supplementary material available at 10.1186/s12888-023-04699-8.

## Introduction

Suicide is a serious health concern globally. Over 700,000 deaths are reported worldwide per annum, with male suicides outnumbering female suicides, and the bulk of suicides occur in low- and middle-income countries (LMICs), where the majority of the world's population live [1]. The global lifetime prevalence of suicidal ideation (SI) and suicide attempts is 9.2% and 2.7%, respectively [1, 2]. Additionally, it is expected that for every individual who dies by suicide, there are more than 20 others who have attempted suicide, and for every person who attempts suicide, there are two to three others who seriously consider it but do not go through with an attempt [3]. The magnitude and seriousness of this public health issue have prompted a significant surge in research investigating SI. Nevertheless, the rate of suicide has not yet diminished [4]. Precise prediction is critical for efficient prevention. As a result, a major focus of suicide research has been on identifying risk factors for SI. Despite this major research concentration, research in this area remains predominately focused on high-income countries, with little research addressing SI in LMICs [5]. The current study, therefore, focuses on the associations between childhood trauma (ChT), depressive symptoms, difficulties in emotion regulation (DER), SI, and gender in an LMIC– Iran.

Suicidal ideation (i.e., having transitory to comprehensive and lingering thoughts of suicide) is recognized as a significant harbinger of subsequent suicide attempts and fatality [6]. SI is among the most significant risk factors for suicide [7], which, without intervention, leads one-third of ideators to attempt suicide [8]. Furthermore, SI has been associated with poor psychological adjustment and functioning, as well as subsequent depressive syndromes [9, 10]. Several theories explain the emergence course of SI. Of relevance to this study, the integrated motivational-volitional model (IMV; [11]) posits that when certain distal risk factors, such as ChT, are present, an individual may be prone to developing SI [12]. Motivational and threat-to-self variables, such as DER [13], are proposed to play a mediating role between this pre-existing susceptibility (i.e., childhood trauma) and risk for SI. Also, depression has been claimed to be one of the strongest predictors of SI (e.g., [14]), which is in close association with both DER [15] and ChT [16].

Childhood trauma, such as parental neglect or physical, sexual, and emotional abuse, are key predisposing risk factors for SI [17]. Several studies have found strong links between childhood sexual, physical, and emotional abuse and SI [18, 19]. According to a recent meta-analysis, all types of ChT are linked to a two- to three-fold greater risk of SI [20]. The links between certain ChT, depressive symptoms, and SI have also been recognized [21]. ChT accelerates the onset of major depressive disorder and worsens its prognosis and severity across the lifespan [22], which is of import as depression is one of the most widely reported risk factors for SI [23].

Moreover, those who have experienced ChT are more likely to have DER, which in turn increases proneness to depressive symptoms [24, 25], which is also regarded as Achilles' heel to SI [26]. According to theories of suicide (e.g., suicide as psychache, Three-Step Theory (3ST)), psychological pain (i.e., psychache) is a major contributor to SI [27, 28] and the capacity for emotion regulation (i.e., the ability to monitor, appraise, and modulate emotional experiences and responses) is an important aspect of managing this pain [29]. There are various theories and frameworks for emotion regulation; nevertheless, integrating research through a shared conceptualization would most probably result in a greater cross-fertilization of outcomes between emotion scientists and the psychopathologies [30]. Thus, in the current study, we chose Gratz and Roemer's [29] emotion regulation and dysregulation paradigm because of its considerable applicability to clinical and psychopathologic contexts. Accordingly, being aware and cognizant of emotions, accepting emotions, having the ability of impulse control and goal-directed behavior in the face of negative emotions, and having the capacity to employ context-pertinent emotion regulation strategies toward individual goals and situational demands, conceptualized as emotion regulation and the lack of each of abovementioned features is defined as difficulties in emotion regulation.

Emotion regulation/dysregulation appears to play an essential role in explaining SI in etiological models (i.e., Interpersonal Theory of Suicide (IPTS) and 3ST) of suicidal processes [28, 31]. In these models, emotion regulation strategies have been regarded as precedents/prohibitors of SI. Both the 3ST and the IPTS imply that perceptions of oneself as detached from others, a burden on others, and high levels of psychological pain are related to failed emotional coping strategies [28, 31]. As a result, studies have frequently indicated that emotional regulation impairments accelerate the development of the SI (e.g., [32]). Also, according to the IMV model, defeat/humiliation perceptions resulting in the sense of entrapment are fundamental to the motivational phase of SI development [12]. Feelings of entrapment as a result of negative self-appraisals reinforce the belief that suicide is the only way out [33].

While several studies have identified risk factors for SI [34–37], further research is needed to examine the direct and indirect associations between theorized risk factors and SI. As noted above, it is theoretically posited that ChT raises the likelihood of SI. Theoretically and empirically, it has been argued that variables, such as emotion regulation and mood disorders, may play a mediating role between this pre-existing susceptibility (i.e., childhood trauma) and SI [13, 34, 38, 39]. There is accumulating empirical support for these theoretical accounts. For instance, Mohammadzadeh et al. [40] found among males who use heroin that, while ChT had no direct effect on SI, ChT was indirectly associated with SI through some emotion regulation difficulties. Roley‐Roberts and colleagues found that facets of emotion dysregulation mediated the associations between child sexual abuse and SI [41]. Hatkevich et al. [42] found among adolescent inpatients that limited access to emotion regulation strategies, difficulties in impulse control, and mood disorder diagnosis were significantly associated with past-year SI. Hatkevich et al. [43] demonstrated that emotional abuse might be differentially related to experiencing limited access to emotion regulation strategies at the level indicative of SI risk. Thus, SI may stem from ChT and, subsequently, the emotional dysregulation [40]. Additionally, Hopfinger and colleagues found that general emotion regulation deficits mediated the association between ChT and both depression severity and depression lifetime persistency [25]. Their findings support the theoretical assumption that DER may play a role in the negative course of depression in those who have experienced ChT.

Therefore, based on theoretical accounts and previous findings that emotion regulation deficits mediate the association between ChT and depression (e.g., [25]), and depression is then associated with SI [44], we propose that it is possible that there is an indirect pathway between ChT and SI through DER and depressive symptoms. Despite accumulating research investigating these associations and theoretically proposed pathways between ChT and SI, an important significant gap in SI research is that much of the research has been conducted in Western cultural contexts and high-income countries. Consequently, there has been an identified need for greater research in LMICs (e.g., [5]). This is particularly important as the majority of suicides occur in LMICs [1]. The current study, therefore, focused on Iran, a country with the highest increase in suicide-related deaths among Islamic countries and the Eastern Mediterranean region [45]. Additionally, research indicates that culture impacts processes such as emotion regulation [46, 47], known to be associated with SI [48], and Iranian researchers have called for greater research exploring the factors contributing to SI and suicide in Iran [49]. Finally, as there is an identified need for national policies and interventions in Iran to target the prevention of suicide, it is critical that risk factors for SI are investigated in Iranian samples [34, 45]. Thus, this study is novel in investigating these associations and pathways that have been found in other cultural contexts in an Iranian sample.

Finally, in the SI literature, gender disparities in suicide susceptibility have not been well addressed [50]. Emerging research aims to highlight possible gender variations in suicide risk vulnerability (i.e., behavior and ideation). In the instance of Iran, higher suicide attempt rates have been found among women, while higher suicide mortality rates have been found among men [49]. Kiadaliri and colleagues [49] suggest several potential explanations for these gender differences, including a) methods of attempting suicide, whereby men in Iran commonly use hanging and firearms, which have higher fatality rates compared with the self-burning method commonly used by women, b) greater psychosocial impact of problems, such as unemployment or retirement, on men compared with women, and c) men adopting coping strategies such as emotional inexpressiveness, lack of help-seeking, risk-taking behavior, violence, and substance use. They also highlight, therefore, the importance of including gender analyses in suicide research, as such information is important for designing and implementing suicide prevention strategies. Moreover, SI has been at the vanguard of suicide research in attempting to understand these gender differences [51]; consequently, investigations targeted at understanding gender differences are critical, and including an examination of gender-specific associations between the study variables are of potential importance.

### Current study

This study aimed to investigate the associations between ChT, depressive symptoms, DER, and SI. We hypothesized that ChT would be positively correlated with depressive symptoms and DER (hypothesis 1). Second, we hypothesized that depressive symptoms and DER would be positively correlated with SI (hypothesis 2). Third, we predicted that there would be a mediated association between ChT and SI through DER and depressive symptoms (hypothesis 3).

## Method

### Participants

A total of 400 individuals were selected from different departments at Chamran University, Iran. Twenty-seven (7%) participants were excluded due to incomplete data. Thus, the final sample included 372 participants. The age range was 18–32, with men having a mean of 20.54 and females having a mean of 20.9 years. Our inclusion criteria were: (1) being aged between 18 and 65, (2) not currently taking psychotropic medications and psychotherapy, and (3) possessing sufficient literality to complete the questionnaire array (i.e., no issues reading or comprehending questionnaires).

### Measures

#### Scale for Suicidal Ideation (SSI; [52])

The SSI, one of the most commonly used measures to assess current suicidal ideation, is a 19-item scale used to assess the current specific attitudes, behaviors, and plans of suicide [53]. Each item is rated on a 3-point Likert scale (0 = *no ideation* to 2 = *strong ideation*). Only patients who express a wish to undertake an active (item no. 4) or passive (item no. 5) suicide attempt are assessed on items 6–19, which are used to screen for attitudes regarding life and death. Total scores are determined by summing the scores on the 19 items, with scores ranging from 0 to 38 and higher scores indicating greater SI. The internal consistency of the SSI is good [53], and the psychometric properties of SSI in Iranian samples have been found to be good (Cronbach's *α* = 0.83) [54]. In the current study, internal consistency was satisfactory (Cronbach's *α* = 0.89). Confirmatory factor analyses showed that the three-factor first-order and one-factor second-order model fitted the data well: χ^2^(116) = 232.478, *p* < 0.01, CFI = 0.99, TLI = 0.99 RMSEA = 0.059, 95% CI [0.096 to 0.109], SRMR = 0.06 (Sup. Fig. 1 and Sup. Table 1).

#### Childhood Trauma Questionnaire (CTQ; [55, 56])

Following the approach of previous researchers, recall of ChT was measured using the Childhood Trauma Questionnaire (CTQ-SF) (e.g., [57]). The CTQ-SF is a 28-item self-report measure that assesses retroactive ChT history in the home. It assesses five main types of ChT: emotional abuse, physical abuse, sexual abuse, emotional neglect, and physical neglect. Each subscale is represented by five items, which are rated on a 5-point Likert scale ranging from *never true (1)* to *very often true (5)*. Each subscale has a score range from 5 to 25 (some items are reverse-coded). The CTQ-SF also includes three questions that comprise a minimization/denial scale that screens for the likelihood of underreporting traumatic experiences. In the current study, we summed the five ChT subscale scores to provide an overall index of the ChT [58]. A cutoff value ≥ 35 for total CTQ scores indicates a significant history of childhood trauma [58]. The psychometric properties of the CTQ-SF are satisfactory [56]. Ebrahimi et al. [59] reported Cronbach's alphas ranging from 0.81-0.97 among Iranian samples. In the current study, internal consistency was good (Cronbach's *α* = 0.90). Confirmatory factor analyses showed that the five-factor first-order and one-factor second-order model fit the data satisfactorily: χ^2^(270) = 629.918, p < 0.01, CFI = 0.97, TLI = 0.97, RMSEA = 0.06, 95% CI [0.054 to 0.066], SRMR = 0.06 (Sup. Fig. 2 and Sup. Table 1).

#### State Difficulties in Emotion Regulation Scale (S-DERS; [60])

Emotion regulation difficulties were assessed by S-DERS, which is a 21-item scale that was created by Lavender et al. [60] to assess the DER in individuals when they try to regulate their emotions in various contexts. Using the original scale as a guide, Difficulties in Emotion Regulation Scale (DERS; [29]), the items were created on a 5-point Likert-style scale ranging from *almost never (1)* to *almost always (5)*. Acceptance, modulation, awareness, and clarity are the four components of the S-DERS. Internal consistency coefficients for the scale in the initial study were 0.86 for the overall score, 0.92 for nonacceptance, 0.85 for modulate, 0.79 for awareness, and 0.65 for clarity. In Iranian samples, the DERS has been found to have good concurrent validity and reliability [61]. In the current research, internal consistency was determined to be good (Cronbach's *α* = 0.88). Internal consistency coefficients were for non-acceptance of emotional responses (Cronbach's *α* = 0.94), lack of emotional knowledge (Cronbach's *α* = 0.86), restricted access to emotional regulation strategies (Cronbach's *α* = 0.87), and emotional clarity (Cronbach's *α* = 0.68). Confirmatory factor analyses of a model with four-factor first-order and one-factor second-order conducted with this study's data indicated good model fit: χ^2^(185) = 898.692, *P* < 0.01, CFI = 0.95, TLI = 0.95, RMSEA = 0.010, 95% CI [0.096 to 0.109], SRMR = 0.06 (Sup. Fig. 3 and Sup. Table 1).

#### Beck Depression Inventory (BDI; [62])

The BDI is a widely used 13-item self-report measure of depressive symptom severity [63] rated on a 4-point scale from 0 (symptom absence) to 3 (severe symptoms). The measure has demonstrated good psychometric properties [52]. The Persian version has high internal consistency (Cronbach's α = 0.89) [64]. In the current study, good internal consistency was found (Cronbach's α = 0.89). Confirmatory factor analyses using the data from this study showed that the three-factor first-order and one-factor second-order model fitted the data satisfactorily: χ^2^(62) = 117.289, *p* < 0.01, CFI = 0.99, TLI = 0.98, RMSEA = 0.049, 95% CI [0.035 to 0.063], SRMR = 0.04 (Sup. Fig. 4 and Sup. Table 1).

### Procedure

This research adhered to the guidelines outlined in the Helsinki Declaration. The Shahid Chamran University of Ahvaz ethics board granted permission for the study to proceed (registration code: 171,107.13980305). All participants provided informed written consent and were informed that taking part in the study was completely voluntary and that they might discontinue at any time. The study took place as an online survey using Google Forms. The participants were asked to answer demographic questions about gender, age, and education before answering the questions in the SSI, CYQ-SF, DERS, and BDI, and no remuneration was provided.

### Analysis strategy

According to the number of predictor variables in the conceptual model, we used G*Power [65] to calculate the minimum sample size required to achieve adequate power (0.80) for a medium effect size (0.30). Our study included 159 men (42.7%) and 213 women (57.3%). Data analysis was conducted using SPSS 28.0.1 statistical software [66] and Mplus 8.8 [67], and five major steps followed:

#### 1^st^ step

All variables were checked for missing values, outliers, and assumptions before conducting the analyses [68]. Lower than 5% of the data was missing data. In the analyses done for our data, list-wise deletion without any data imputation was utilized. The questionnaire subscales showed no skewness, and the assumption of normalcy was verified. There were no transformations carried out due to the proper size of the sample.

#### 2^nd^ step

The internal consistencies of the SSI, CTQ, DERS, and BDI were evaluated using Cronbach's alpha [69, 70]. Here, an appropriate level of item internal consistency was defined as a correlation value of 0.70 or greater (See; [71]). All the measures utilized in this study were subjected to a second-order factor analysis to confirm the theoretical construct's division into a predetermined number of subcomponents.

#### 3^rd^ step

We employed CFA implementing Weighted Least Squares Mean–Variance Adjusted (WLSMV) to examine a priori models of the scales’ factorial validity [72, 73]. Then the following statistical tests and goodness-of-fit indices were employed to evaluate the proposed models: Chi-square/degree of freedom CMIN/DF—where values < 3.0 indicate good fit [74], the Root Mean Square Error of Approximation (RMSEA ≤ 0.06 suggests good fit) [74, 75], the Comparative Fit Index (CFI) where coefficients > 0.95 indicate good fit; [76], the Tucker-Lewis index (TLI), coefficients > 0.95 indicate good fit [77]. Moreover, the Satorra-Bentler scaled chi-square test statistic was used to correct the multivariate skewness in our data and the fit indices [75].

#### 4^th^ step

**Fig. 1:**
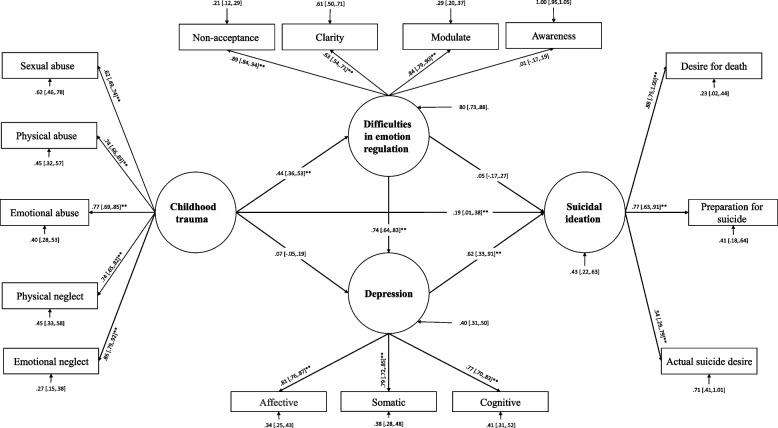
Structural equation modeling of pathways from childhood trauma to suicide ideation: mediating through difficulties in emotion regulation and depressive symptoms

**Fig. 2:**
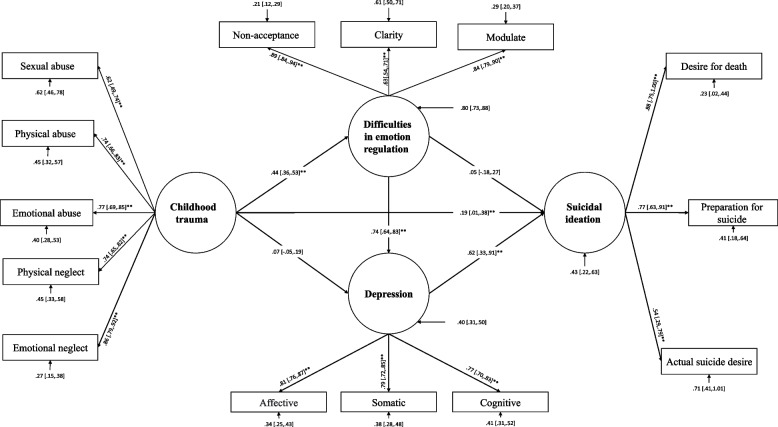
Structural equation modeling of pathways from childhood trauma to suicide ideation: mediating through emotion regulation difficulties and depressive symptoms, after taking the awareness (observed index) out of the indicators of difficulties in emotion regulation

**Fig. 3:**
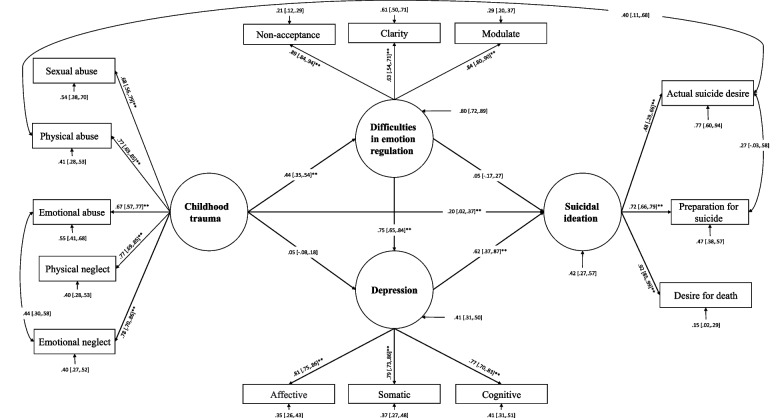
Structural equation modeling of pathways from childhood trauma to suicide ideation: mediating through emotion regulation difficulties and depressive symptoms when excluding awareness (observed index) from the indicators of difficulties in emotion regulation and according to the modification indices, error covariances were freed to correlate: 1- emotional neglect with emotional abuse; 2- physical abuse with actual suicide desire; 3- actual suicide desire with preparation for suicide

Suicide observed indicators (i.e., desire for death, preparation for suicide, actual suicide desire) were entered into Model 1 as exogenous variables, childhood trauma’s observed indicators (i.e., sexual abuse, physical abuse, emotional abuse, emotional neglect, physical neglect) as endogenous variables and depressive symptoms and DER as mediators. Depressive symptoms is regarded as a dependent variable of DER in the current conceptual model specification (Fig. 1). In model 2, according to modification indices in factors with poor fit index, they (i.e., awareness) have been excluded from the model (See Fig. 2). In model 3, three error covariances, namely emotional abuse with emotional neglect, physical abuse with actual suicide, and actual suicide with preparation, were freed to correlate (Fig. 3).

#### 5^th^ step

In Mplus 8.8, the MODEL INDIRECT command is used to obtain classic indirect, direct, and total effects as well as their standard errors, all effects that are utilized in the traditional mediation research [78]. Of note, in steps 4 and 5, using maximum likelihood estimation (MLR) with the robust standard errors [73], the mediator model was evaluated. The bias-corrected bootstrap approach was used to test indirect effects (100,000 replications with 95% confidence intervals [CIs]). Bootstrapping is implemented to analyze the indirect effects' significance [79].

## Results

The bivariate correlations between ChT, depressive symptoms, DER, and SI are presented in Table 1. As evident in Table 1, all bivariate correlations were significant, which showed that all endogenous and exogenous indicators were associated (except physical abuse and cognitive subscale of depression, r = 0.07). As a result, it can be said that the conceptual model that has been provided has solid conceptual and statistical underpinnings for investigating the mediation model. (Figs. 1, 2 and 3 and Table 2).

**Table 1:** Means, standard deviations, and bivariate correlations between childhood trauma, emotion regulation difficulties, depression, and suicidal ideation

**Variable**	**M**	**SD**	1	2	3	4	5	6	7	8	9	10	11	12	13	14	15	16	17	18	19
1. **Childhood Trauma**	33.04	10.79	1																		
2. SA	5.92	2.02	0.70^**^	1																	
3. PhA	6.43	2.32	0.81^**^	0.58^**^	1																
4. EA	8.1	3.75	0.84^**^	0.39^**^	0.57^**^	1															
5. PhN	6.43	2.32	0.80^**^	0.58^**^	0.59^**^	0.54^**^	1														
6. EN	7.38	2.95	0.87^**^	0.44^**^	0.64^**^	0.72^**^	0.61^**^	1													
7. **Difficulties in Emotion Regulation**	52.27	16.61	0.36^**^	0.28^**^	0.23^**^	0.35^**^	0.27^**^	0.30^**^	1												
8. NA	16.91	7.8	0.38^**^	0.30^**^	0.23^**^	0.35^**^	0.30^**^	0.31^**^	0.89^**^	1											
9. C	5.31	2.01	0.22^**^	0.15^**^	0.09	0.27^**^	0.19^**^	0.17^**^	0.66^**^	0.57^**^	1										
10. M	18.12	6.76	0.41^**^	0.32^**^	0.29^**^	0.39^**^	0.31^**^	0.34^**^	0.89^**^	0.76^**^	0.56^**^	1									
11. A	18.02	4.7	0.18^**^	0.16^**^	0.11^*^	0.14^**^	0.19^**^	0.12^*^	0.11^*^	0.23^**^	0.11^*^	0.17^**^	1								
12. **Depression**	7.50	6.97	0.35^**^	0.27^**^	0.18^**^	0.35^**^	0.31^**^	0.30^**^	0.60^**^	0.63^**^	0.44^**^	0.60^**^	0.24^**^	1							
13. Af	2.14	2.94	0.36^**^	0.29^**^	0.25^**^	0.31^**^	0.33^**^	0.29^**^	0.53^**^	0.58^**^	0.34^**^	0.50^**^	0.18^**^	0.88^**^	1						
14. S	2.74	2.41	0.30^**^	0.21^**^	0.12^*^	0.32^**^	0.26^**^	0.27^**^	0.52^**^	0.57^**^	0.41^**^	0.53^**^	0.27^**^	0.85^**^	0.62^**^	1					
15. Co	2.62	2.69	0.25^**^	0.18^**^	0.07	0.29^**^	0.21^**^	0.23^**^	0.50^**^	0.50^**^	0.40^**^	0.52^**^	0.20^**^	0.87^**^	0.62^**^	0.64^**^	1				
16. **Suicidal Ideation**	3.10	5.51	0.39^**^	0.20^**^	0.37^**^	0.37^**^	0.34^**^	0.29^**^	0.45^**^	0.49^**^	0.27^**^	0.43^**^	0.14^**^	0.58^**^	0.60^**^	0.38^**^	0.50^**^	1			
17. DD	1.61	2.08	0.38^**^	0.22^**^	0.31^**^	0.36^**^	0.33^**^	0.30^**^	0.49^**^	0.53^**^	0.34^**^	0.47^**^	0.18^**^	0.63^**^	0.58^**^	0.48^**^	0.58^**^	0.84^**^	1		
18. P	1.05	2.56	0.33^**^	0.14^**^	0.31^**^	0.33^**^	0.30^**^	0.23^**^	0.40^**^	0.42^**^	0.21^**^	0.39^**^	0.11^*^	0.50^**^	0.53^**^	0.32^**^	0.42^**^	0.95^**^	0.69^**^	1	
19. AS	0.45	1.49	0.37^**^	0.20^**^	0.41^**^	0.32^**^	0.30^**^	0.28^**^	0.31^**^	0.33^**^	0.16^**^	0.31^**^	0.10^*^	0.40^**^	0.48^**^	0.22^**^	0.31^**^	0.89^**^	0.60^**^	0.83^**^	1

*SA=* Sexual Abuse, *PhA=* Physical Abuse, *EA= *Emotional Abuse, *PhN=* Physical neglect, *EN=* Emotional neglect, *NA=* Non-acceptance, *C=* Clarity, *M=* Modulate, *A=* Awareness, *Af=* Affective, *S=* Somatic, *Co=* Cognitive, *DD=* Desire for death, *P=* Preparation, *AS=* Actual Suicide

^∗^*p* < 0.05

^∗∗^*p* < 0.01

**Table 2:** Modification indices for the mediated model of childhood trauma and suicidal ideation, difficulties in emotion regulation, and depression

Model	*χ*^*2*^	*df*	*χ*^*2*^/df	CFI	TLI	RMSEA	SRMR	Base model	ΔS-Bχ^2^(Δdf)
M_1_	391.90	84	4.66	0.89	0.86	0.100(0.090—0.110)	0.60	-	-
M_2_	339.91	71	4.79	0.90	0.87	0.101 (0.091—0.112)	0.057	M_1_	51.99^***^(13)
M_3_	216.86	68	3.19	0.95	0.93	0.077(0.066—0.089)	0.049	M_1_	175.04^***^(16)

M_1_ = Structural equation modeling of pathways from childhood trauma to suicide ideation: mediating through difficulties in emotion regulation and depressive symptoms, M_2_ = M_1_ + removingawareness (observed index) from the indicators of difficulties in emotion regulation. M_3_ = M_2_ + the following error covariances were freed to correlate: 1- emotional neglect with emotional abuse; 2- physical abuse with actual suicide desire; 3- actual suicide desire with preparation for suicide. *χ*^*2*^ = Chi-square, df = degrees of freedom, *χ*^*2*^/df = Normal Chi-square, *TLI =* Tucker–Lewis Index, *CFI =* Comparative Fit Index, *SRMR =* Standardized Root Mean Square Residual, *RMSEA =* Root Mean Square Error of Approximation, Δ*χ*^*2*^ =Difference between minus twice log likelihoods between the full and the nested models

^∗^*p* < 0.05

^∗∗^*p* < 0.01

^∗∗∗^*p* < 0.001

### Mediation analyses

The goodness-of-fit results for all nested-mediated models are presented in Table 2. A theory-driven specified model (M_1_ in Table 2 and Fig. 1; S-B χ^2^ = 391.90, CFI = 0.89, TLI = 0.86, and RMSEA = 0.10 (95% CI [0.09, to 0.11]) failed to meet the previously specified fitting criteria. As a result, Model 1 was modified into Model 2, which was able to reasonably fit the data. Though, removing the nonsignificant indicator of DER (awareness; α = 0.008, bootstrapping 95% CI = [-0.17 to 0.19]) enhanced the initial model's fitness (M_2_ in Table 2 and Fig. 2). Despite this, it did not meet all criteria (M_2_ in Table 2: S-B χ^2^ = 339.91, CFI = 0.90, TLI = 0.87, and RMSEA = 0.10 ( 95% CI = [0.09 to 0.11]). Following a closer examination of modification indices for error covariances, specific error covariances for latent variables of interest were freed to correlate sequentially (M_3_ in Table 2). To evaluate whether the fit indices improved after freeing error covariance between emotional neglect with emotional abuse (β = 0.44, bootstrapping 95% CI = [0.27 to 0.56]), physical abuse with actual suicide intention (β = 0.40, bootstrapping 95% CI = [0.14 to 0.67]) and actual suicide intention with preparation (β = 0.27, bootstrapping 95% CI = [0.04 to 0.64]) have been freed to correlate, respectively. Freeing error covariances significantly improved the fitting of the model, as shown in Table 2 (M_3_: CFI = 0.95, TLI = 0.93, and RMSEA = 0.07 95% CI = [0.06 to 0.09]). Sequentially incorporating error covariance terms improved the model's fitness with the data. The final two models were evaluated according to the parsimony principle (M_2_ & M_3_: Δ S-B χ^2^ = 175.04; *P* < 0.001). According to an evaluation of fitness indices among the nested models, the third model (M_3_) (Table 2 and Fig. 3) was found to be the most effective modification of the proposed, mediated model. Results of Table 3 show a significant direct association between ChT and DER (β = 0.44, bootstrapping 95% CI = [0.35 to 0.54]), depressive symptoms, and SI (β = 0.62, bootstrapping 95% CI = [0.37 to 0.87]), DER and depressive symptoms (β = 0.75, bootstrapping 95% CI = [0.65 to 0.84]) and finally between ChT and SI (β = 0.20, bootstrapping 95% CI = [0.02 to 0.37]). According to Table 4 our findings indicate that DER is mediating the association between ChT and depressive symptoms (β = 0.33, bootstrapping 95% CI = [0.16 to 0.32]). Also, there is a significant indirect effect between ChT and SI (β = 0.26, bootstrapping 95% CI = [0.31 to 0.60]). It is also observed that the association between ChT and SI is mediated through DER and depressive symptoms (β = 0.20, bootstrapping 95% CI = [0.12 to 0.32]).

**Table 3:** Standardized direct effects of childhood trauma, difficulties in emotion regulation, depression, suicidal ideation

Paths	Direct effect	*p*	95% CI
Childhood Trauma** → **Difficulties in Emotion Regulation	0.44	0.001	[0.35 0.54]
Childhood Trauma** → **Depression	0.05	0.43	[-0.08 0.18]
Depression → Suicidal ideation	0.62	0.001	[0.37 0.87]
Difficulties in Emotion Regulation → Suicidal ideation	0.05	0.65	[-0.17 0.27]
Difficulties in Emotion Regulation → Depression	0.75	0.001	[0.65 0.84]
Childhood Trauma → Suicidal Ideation	0.20	0.03	[0.02 0.37]

*CI:* Confidence Intervals

**Table 4:** Indirect standardized effects of childhood trauma, difficulties in emotion regulation, and depression on suicidal ideation using the bootstrap method

	Estimation	SE	T-value	P	95% Confidence Intervals
Childhood Trauma → Suicidal Ideation					
Total Effect	0.46	0.08	4.05	0.001	[0.31 to 0.60]
Total Indirect	0.26	0.05	4.89	0.001	[0.16 to 0.37]
Childhood Trauma → Depression → Suicidal Ideation					
Indirect Effect	0.03	0.04	0.75	0.46	[-0.04 to 0.013]
Childhood Trauma → Difficulties in Emotion regulation → Suicidal Ideation					
Indirect Effect	0.02	0.05	0.46	0.65	[-0.07 to 0.12]
Childhood Trauma → Difficulties in Emotion Regulation → Depression					
Indirect Effect	0.33	0.04	8.05	0.001	[0.16 to 0.32]
Childhood Trauma → Difficulties in Emotion regulation → Depression → Suicidal Ideation					
Indirect Effect	0.20	0.05	4.10	0.001	[0.12 to 0.32]

## Discussion

The aim of the present study was to explore the mediation pathways from ChT to SI through depressive symptoms and DER. In support of hypothesis 1, ChT had significant positive associations with depressive symptoms and DER. Second, depressive symptoms and DER were significantly associated with SI (hypothesis 2). Extant literature shows that the association found between ChT and depressive symptoms is consistent with the extensive previous literature (e.g., [76]). In the context of ChT, parents can be emotionally unavailable, children can experience chronic interpersonal stress (rejection and stifled social support), and children can form insecure attachments, factors all associated with adulthood depression [80, 81]. Our finding that depressive symptoms were directly associated with SI aligns with strong evidence indicating that depression is one of the most widely reported risk factors for SI [, , , 3, 23, 44, 82]. Additionally, ChT had a significant direct association with DER. Kim and Cicchetti [83], in a longitudinal study, reported ChT, specifically emotional neglect and physical and sexual abuse, were related to difficulties in emotion regulation. Individuals with ChT, when compared to those without a history of ChT, used less adaptive emotion regulation strategies. Thus, ChT appears to play a crucial role in developing poor emotion regulation strategies [84].

Regarding hypothesis 3, our findings revealed interesting mediation effects. First, we found that neither DER nor depressive symptoms have mediated the association between ChT and SI solely. These results seem counterintuitive at first glance. Nevertheless, when we inspected more closely, we discovered that DER and depressive symptoms function together (in a sequential path) in the theory-adaptive sequence to mediate the association between ChT and SI. This finding reveals a more complex, comprehensive, and interpretative association between distal and proximal associations between ChT and SI. Our model results indicate that ChT, without taking into account DER, is not explaining depressive symptoms and SI. But in a more composite and inclusive panorama, ChT can contribute to depressive symptoms and then SI in the mere existence and presence of DER. This finding fits within the framework of transdiagnostic theories of emotion regulation, namely the heuristic model of emotion regulation [85]. The authors postulated that a combination of proximal (endophenotype) and distal (traumatic experience, genetic proneness) factors would cause inflexible emotion regulation (e.g., rumination) to initiate a variety of externalizing and internalizing disorders. In our study’s setting, ChT can act as the distal factor leading to more proximal risk factors (i.e., DER) and finally lead toward depressive symptoms and SI as a manifestation of depression or as an experiential avoidance strategy [86] used to shy away from all psychache [27, 87] imposed by ChT and depressive states. This finding immaculately confirms Mohammadzadeh et al.s’ [40] findings in which they found that ChT was not directly linked to SI; it was indirectly linked to SI through DER. Intriguingly, in a longitudinal study with a sizable sample (i.e., 5423), Wu et al. [39] found that in a mediation model, emotion reactivity (an index of DER) leads to depression, and depression leads to SI, which is precisely consistent with our findings and model. Consistently and particularly, Hatkevich et al. [43] researched different forms of childhood abuse and found that compared to other forms of abuse and neglect, emotional abuse may be more strongly associated with having restricted access to emotion regulation strategies during adolescence, which is a condition suggestive of a higher risk of SI. Demirci also reported associations between childhood sexual abuse, DER, and diverse psychiatric conditions [88]. Other studies also are indicative of associations between ChT, depression, and suicidal behavior [89, 90]. Individuals who have experienced ChT have a lower activation threshold as well as a broader spectrum of internal and external triggers for SI. Indeed, a trauma-related image or thought can precipitate a crisis, operate as a forerunner, and cause unpleasant affective states and emotions that can lead to suicidal behavior [91]. Hatkevich et al. [42] also observed that DER and a diagnosis of a mood disorder were all linked to SI in the previous year. As parents/caregivers play a critical role in structuring, elaborating, and regulating a child's emotions [92], children exposed to trauma (e.g., physical abuse, emotional neglect) may, in turn, perceive the world as unpredictable and threatening and others (particularly parents) are not emotionally available to provide required structure and regulation of emotions [83]. This unavailability would increase the DER and also increases the possibility of psychopathology, peer rejection [93], and interpersonal difficulties [94], leading to self-mutilative thoughts and behaviors, including SI.

Historically, identifying those most vulnerable to SI and attempts has been challenging; the huge number of possible risk variables has made such predictions inaccurate [95]. Clinicians need to have a thorough awareness of the risk factors for suicide and how they combine to increase the risk of suicide. The present study's findings have important therapeutic relevance to clinical and clinical analogue populations since the variables assessed (i.e., depressive symptoms, DER, and ChT) have been shown to play a role in SI. This is the first time such variables and pathways have been investigated in Iran. This is of relevance in Iran, where the need for policies and interventions targeting the prevention of suicide has been identified [34]. Thus, the findings indicate that such targets may have applicability in Iran and may help to assess suicide risk levels more accurately.

### Limitations

Alongside its strength, our study has some limitations. Self-reports of emotion regulation may not always be accurate [96]. Second, the cross-sectional design means causality cannot be inferred. While this study was novel in that it investigated these associations for the first time, commonly observed in previous cross-sectional research conducted in high-income countries, in Iran, further studies are now needed using longitudinal designs. Third, more objective and context-specific measures of emotion regulation (e.g., ecological momentary assessment) [97] should be used in future studies, and future replication of longitudinal studies with diverse types of suicidal behavior spectrum is needed in a range of cultural contexts (Cf. [98, 99]). Also, measures of ChT were retrospective self-reports. Finally, because the sample sizes would start to shrink when we subdivided the sample into age and/or gender subgroups, the present study data did not run the multigroup-mediated analysis across gender. In the gender case, the sample size would be down to 159 for males. Such small sample sizes lack sufficient power to detect any invariance. In a multigroup analysis, 200 participants should be considered for hypotheses involving full and strong invariance. As reported by Meade and Bauer [100], There is low power to detect invariance in samples of < 400. Therefore, due to the non-sufficient sample size and, consequently, low power, invariance analysis of the finally selected mediated model of pathways from ChT to SI was not probed [101].

## Conclusions

The current study examined direct and indirect associations between SI-related variables in the proposed conceptual model, and there was a significant indirect effect of ChT on SI through DER and depressive symptoms. Despite that our study was a cross-sectional study, it was able to cost-efficiently replicate the findings of large-scale longitudinal investigations (e.g., [38, 39]). There are important clinical implications that may be inferred from the mediation path that our study identified. Nearly 40% of participants in the World Mental Health (WMH) initiative in 21 countries reported having traumatic childhood experiences [102]. Concerning this high prevalence, concentrating on the emotion regulation intervention (e.g., [103]) in particular can be a sensible preventive strategy that may buffer against the enduring effects of ChT while also reducing depressive symptomatology and, ultimately, diminishing SI and behavior.

## Supplementary Information

**Additional file 1: Supplementary Figure 1.** Three first-order and one second-order confirmatory factor analyses of the Scale for Suicidal Ideation (SSI). **Supplementary Figure 2.** Five first-order and one second-order confirmatory factor analyses of the Childhood Trauma Questionnaire (CTQ). **Supplementary Figure 3.** Four first-order and one second-order confirmatory factor analyses of State Difficulties in Emotion Regulation Scale (S-DERS). **Supplementary Figure 4.** Three first-order and one second-order confirmatory factor analyses of the Beck Depression Inventory (BDI). **Supplemental Table 1.** Measurement model of childhood trauma, suicidal ideation, difficulties in emotion regulation, and depression.

## Data Availability

The datasets used and/or analyzed during the current study are available from the corresponding author upon reasonable request.

## Abbreviations

ChT: Childhood trauma
DER: Difficulties in emotion regulation
SI: Suicidal ideation
3ST: Three-step theory
IPTS: Interpersonal theory of suicide
