# Supplementary material for: Pathways from childhood trauma to suicidal ideation: mediating through difficulties in emotion regulation and depressive symptoms
Source: BMC Psychiatry. 2023 Apr 28;23:295. doi: 10.1186/s12888-023-04699-8 (PMC10148382; doi:10.1186/s12888-023-04699-8)
Supplement: Supplementary file 1 — Additional file 1: Supplementary Figure 1. Three first-order and one second-order confirmatory factor analyses of the Scale for Suicidal Ideation (SSI). Supplementary Figure 2. Five first-order and one second-order confirmatory factor analyses of the Childhood Trauma Questionnaire (CTQ). Supplementary Figure 3. Four first-order and one second-order confirmatory factor analyses of State Difficulties in Emotion Regulation Scale (S-DERS). Supplementary Figure 4. Three first-order and one second-order confirmatory factor analyses of the Beck Depression Inventory (BDI). Supplemental Table 1. Measurement model of childhood trauma, suicidal ideation, difficulties in emotion regulation, and depression. [file 12888_2023_4699_MOESM1_ESM.docx]

**Supplementary Figure 1.**

*Three first-order and one second-order confirmatory factor analyses of* *the* *Scale for Suicidal Ideation (SSI)*


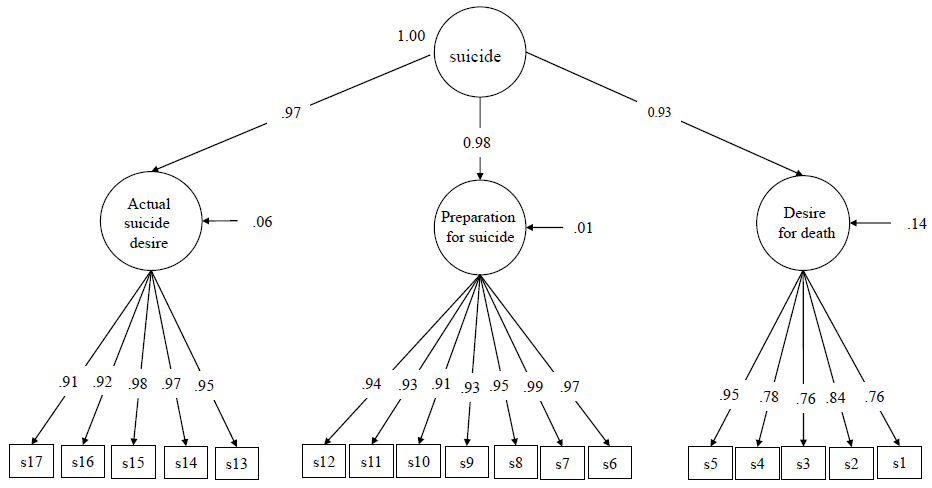


**Supplementary Figure 2.**

*Five first-order and one second-order confirmatory factor analyses of the Childhood Trauma Questionnaire (CTQ)*


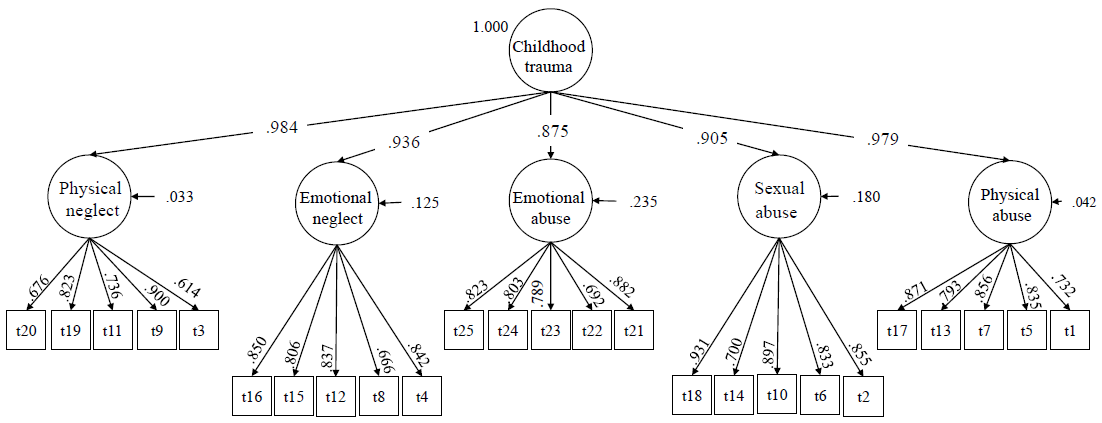


**Supplementary Figure 3.**

*Four first-order and one second-order confirmatory factor analyses of State Difficulties in Emotion Regulation Scale*


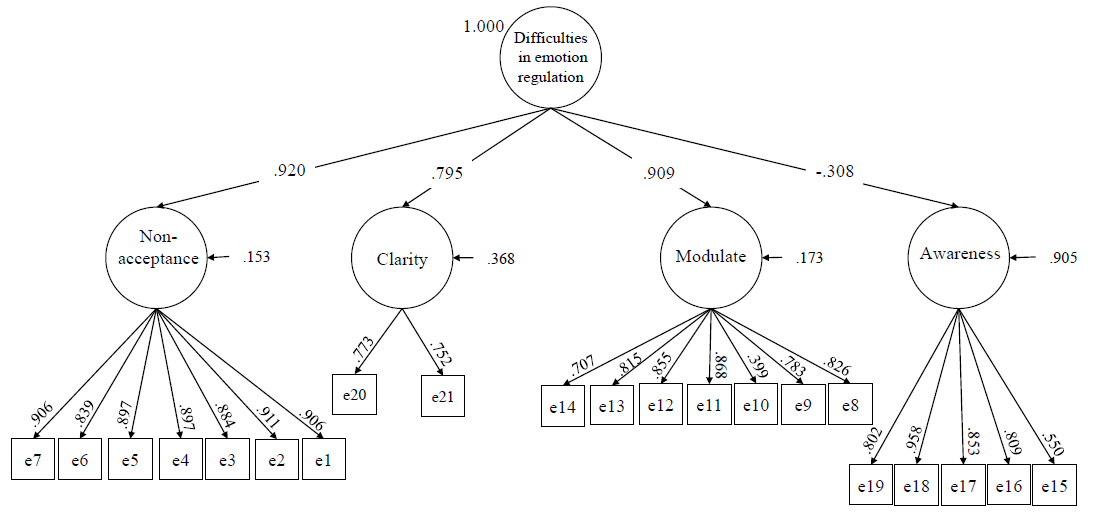


**Supplementary Figure 4.**

*Three first-order and one second-order confirmatory factor analyses of the Beck Depression Inventory (BDI)*


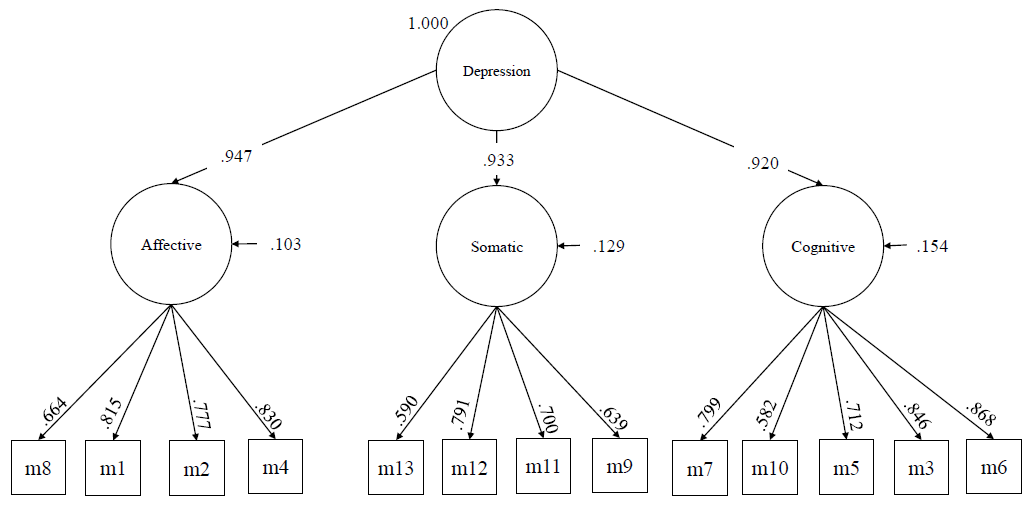


**Supplemental Table 1**.

*Measurement model of childhood trauma, suicidal ideation, difficulties in emotion regulation, and depression*

| **Latent** | **Indicator** | **Parameter estimation** | **SE** | **T value** | **p-value** | **95% Confidence Intervals** |
| --- | --- | --- | --- | --- | --- | --- |
| Childhood Trauma | Sexual Abuse | .68 | .06 | 11.44 | .0001 | [.56 .79] |
| Childhood Trauma | Emotional Abuse | .67 | .05 | 13.21 | .0001 | [.57 .77] |
| Childhood Trauma | Emotional Neglect | .78 | .04 | 18.86 | .0001 | [.69 .85] |
| Childhood Trauma | Physical Neglect | .77 | .04 | 18.68 | .0001 | [.69 .85] |
| Childhood Trauma | Physical Abuse | .77 | .04 | 18.46 | .0001 | [.69 .85] |
| Depression | Affective | .81 | .03 | 29.10 | .0001 | [.75 .86] |
| Depression | Somatic | .79 | .03 | 24.05 | .0001 | [.73 .86] |
| Depression | Cognitive | .77 | .03 | 22.72 | .0001 | [.70 .83] |
| Difficulties in Emotion regulation | Non-acceptance | .89 | .02 | 38.17 | .0001 | [.84 .94] |
| Difficulties in Emotion regulation | Modulation | .84 | .03 | 33.14 | .0001 | [.80 .90] |
| Difficulties in Emotion regulation | Clarity | .63 | .04 | 14.47 | .0001 | [.54 .71] |
| Suicidal Ideation | Desire for Death | .92 | .04 | 24.42 | .0001 | [.85 .99] |
| Suicidal Ideation | Actual Suicide Intention | .48 | .09 | 5.16 | .0001 | [.29 .66] |
| Suicidal Ideation | Preparation | .72 | .03 | 21.27 | .0001 | [.66 .79] |
